# Electron transport chains as a window into the earliest stages of evolution

**DOI:** 10.1073/pnas.2210924120

**Published:** 2023-08-14

**Authors:** Aaron D. Goldman, Jessica M. Weber, Douglas E. LaRowe, Laura M. Barge

**Affiliations:** ^a^Department of Biology, Oberlin College, Oberlin, OH 44074; ^b^Blue Marble Space Institute of Science, Seattle, WA 98154; ^c^NASA Jet Propulsion Laboratory, California Institute of Technology, Pasadena, CA 91109; ^d^Department of Earth Sciences, University of Southern California, Los Angeles, CA 90089

**Keywords:** ATP synthase, early evolution, last universal common ancestor, membrane bioenergetics, origin of life

## Abstract

The origin and early evolution of life is generally studied under two different paradigms: bottom up and top down. Prebiotic chemistry and early Earth geochemistry allow researchers to explore possible origin of life scenarios. But for these “bottom–up” approaches, even successful experiments only amount to a proof of principle. On the other hand, “top–down” research on early evolutionary history is able to provide a historical account about ancient organisms, but is unable to investigate stages that occurred during and just after the origin of life. Here, we consider ancient electron transport chains (ETCs) as a potential bridge between early evolutionary history and a protocellular stage that preceded it. Current phylogenetic evidence suggests that ancestors of several extant ETC components were present at least as late as the last universal common ancestor of life. In addition, recent experiments have shown that some aspects of modern ETCs can be replicated by minerals, protocells, or organic cofactors in the absence of biological proteins. Here, we discuss the diversity of ETCs and other forms of chemiosmotic energy conservation, describe current work on the early evolution of membrane bioenergetics, and advocate for several lines of research to enhance this understanding by pairing top–down and bottom–up approaches.

The earliest fossil and phylogenetic evidence suggests that life has been present on Earth for at least 3.5Ga (1–5). But how life began remains one of the most challenging research questions. Since the first prebiotic chemistry experiments 70 y ago (6) in which amino acids were produced in a simulated early Earth environment, prebiotic chemists have shown that a wide range of biomolecules can be formed abiotically: such as amino acids (7–9), carbohydrates (10, 11), nucleobases (12, 13), nucleotides (14, 15), and membrane forming amphiphilic compounds (16, 17)—as well as more complex molecules such as depsipeptides (18–20) and nucleotide oligomers and supramolecular assemblies (21–23). While many problems of prebiotic chemistry remain to be investigated, the field as a whole has produced a substantial abiotic chemical inventory that can be used to develop plausible models for the origin of life.

In concert with this prebiotic chemistry research, early Earth geochemists have proposed a number of environments that may have been favorable to prebiotic reactions, such as hydrothermal systems on land or the seafloor (24–28), iron-sulfur mineral surfaces (29, 30), clays (31, 32), and ice (8, 33), among others. While no environment has been shown to provide the perfect mixture of thermodynamic drive, molecular stabilization, and the production of a comprehensive suite of protobiomolecules, several of the proposed origin of life environments suggest that these details are not out of reach. Furthermore, it is also possible that different geochemical environments may have facilitated separate stages of the origin of life (34).

The combined success of prebiotic chemistry and early Earth geochemistry, therefore, presents multiple imperfect, but feasible pathways toward an origin of life. However, prebiotic chemistry theories are only hypothetical, not historical—they demonstrate how an origin of life is possible, but cannot determine which among equally plausible hypotheses represents the actual origin of life as it occurred on the early Earth. Thus, prebiotic chemistry and geochemistry research provide evidence about how potential origins of life *may* have occurred, rather than how the origin of life *did* occur (35).

Evolutionary phylogenetic analysis, on the other hand, is fundamentally a historical science that uses extant gene or protein sequences to infer the family history of those genes or proteins and reconstruct their ancestral states. Many studies over the last two decades have attempted to reconstruct the proteome of the last universal common ancestor (LUCA) of all organisms alive today (36). Though large-scale LUCA proteome reconstructions may produce incorrect predictions due to horizontal gene transfer, gene loss, and incomplete taxonomic sampling (37, 38), these LUCA proteome studies tend to infer that the same general categories of molecular functions and biological systems were established in the LUCA (36), and the consensus predictions among multiple studies may be more accurate than any one individual study (38).

Taken together, the LUCA appears to represent a population of organisms with many of the molecular systems that are seen in extant life, including protein-mediated metabolism (39, 40), cellular organization (41, 42), and a genetic system consisting of a DNA genome (43–45), RNA transcripts (46), the translation machinery (47–52), and the canonical genetic code (53–56). Given the results of proteome reconstructions and other phylogenetic analyses of ancient protein families, the LUCA appears to represent a stage in evolution well past the origin of life (36).

A small number of protein families allow researchers to use standard phylogenetic methods to reconstruct evolutionary events prior to the LUCA with a high degree of confidence (57, 58). These protein families resulted from duplications that occurred after the origin of life but before the time of the LUCA yielding two ancestral nodes that can both be associated with the LUCA, or “universal paralogs”. The branch connecting these LUCA nodes, therefore, represents an evolutionary event prior to the LUCA. Most of these universal paralog families are components of the translation system, e.g., the family containing the translation regulatory proteins Elongation Factor tu, Elongation Factor G, and Initiation Factor (47), as well as several families of aminoacyl transfer ribonucleic acid synthetases (48, 49). Other universal paralog families encode proteins with membrane-related functions such as the SRP membrane targeting system (59), subunits of the adenosine triphosphate (ATP) synthase complex (60), and membrane-spanning ATP-binding cassette transporter (ABC-transporters) (58). In addition to ATP synthase, other metabolism-associated universal paralog families include aspartate aminotransferase, which is involved in amino acid metabolism and gluconeogenesis, and acetyl-CoA synthase, which is one method of generating acetyl-CoA to be used in the citric acid cycle (58).

While universal paralog families are valuable to understanding pre-LUCA evolutionary history, they are also rare. In addition to the protein family being old, its function must also require that both paralogs be maintained within genomes throughout subsequent evolution. Initially, these universal paralog families were used to identify the position of the root on the tree of life (59, 60). More recently, ancestral sequence reconstructions have been used to characterize the functions of the ancient pre-LUCA ancestral proteins (41, 47, 48). These reconstructions of pre-LUCA ancestral proteins all depict relatively complex, multidomain proteins that use all or nearly all of the 20 canonical biological amino acids (48, 49). This observation suggests that even these ancestors, which so far are the most ancient proteins that can be reconstructed by phylogenetic analysis, are too complex to represent stages at or immediately following the origin of life.

Research on the origin and early evolution of life therefore comprises two areas of understanding that in practice make little contact with one another (Fig. 1). We understand some of the chemistry and geochemical settings that could have led to an origin of life and we can also infer details about early organisms through the most ancient protein families. In practice, however, these two fields of research remain largely disconnected. Here, we argue that research on the origin and early evolution of life would greatly benefit from focusing on areas where prebiotic geochemistry and phylogenetic analysis overlap. In particular, we argue that electron transport chains (ETCs) and related modes of energy conservation offer a prime target for such research. ETCs, and the ATP synthase motor complex that they support, represent a ubiquitous and diverse strategy for converting redox potential into ATP in extant life. The ATP synthase enzyme is a membrane-spanning multiprotein complex that uses the motive force of proton gradients (61), or in some cases sodium gradients (62), to synthesize ATP from adenosine diphosphate (ADP) and inorganic phosphate. The ATP synthase enzyme is ubiquitous across the tree of life (63) and its evolutionary history predates the LUCA (60).

**Fig. 1.**
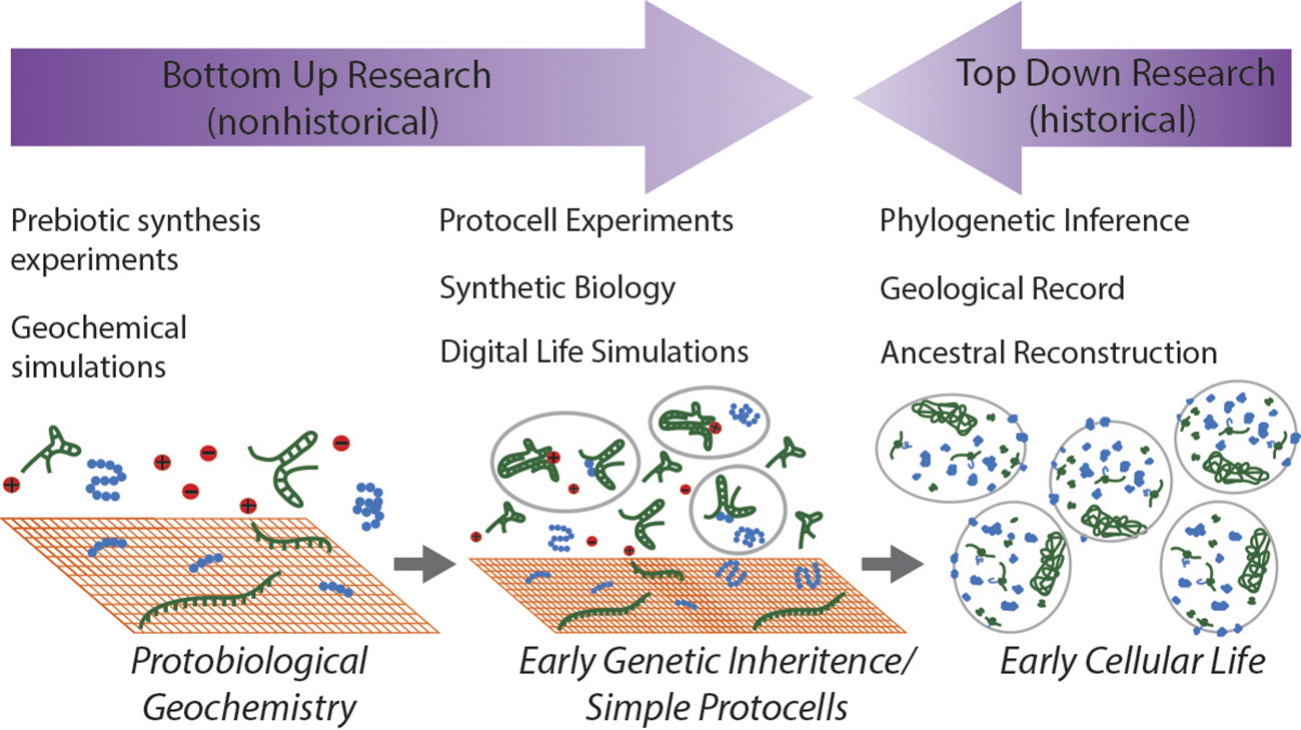
A general progression of the origin and early evolution of life and the scientific approaches that address each stage. The Protobiological Geochemistry stage, sometimes referred to as protometabolism or chemical evolution, depicts abiotic chemistry in a geochemical context that produced complex organic compounds and prebiotic macromolecules. The Early Genetic Inheritance/Simple Protocells stage depicts an early lifeform with a simple genetic system such as proposed by the RNA World hypothesis encapsulated within membranes that form and divide spontaneously. The Early Cellular Life stage depicts organisms with the level of complexity similar to that of the LUCA. While this final stage may be studied through so-called top–down methods, earlier stages may only be accessed through nonhistorical bottom–up approaches.

The proton or ion gradients that power ATP synthases are nearly always (although not always) produced by ETCs. ETCs receive electrons from reduced cofactors generated in other metabolic pathways and pass the electron through a series of membrane-bound enzyme complexes, using the change in reduction potential to pump protons across the membrane. Despite the ubiquity of ATP synthase enzymes across the tree of life, they are supported by many different types of ETCs that are, themselves, determined by both the mode of metabolism of the organism and the evolutionary history of its taxonomic lineage (Fig. 2). Despite this diversity, recent top–down research has identified common and evolutionarily homologous features that are shared across various ETCs. At the same time, advances in prebiotic chemistry and protocell experiments have produced abiotic analogs to components of ETCs. Significant evolutionary change must have taken place between the origin of life and the stage of evolution represented by the LUCA. But if a protein family has been conserved over 3.5-4 billion years of evolution since the time of the LUCA, it is not far-fetched to also assume that the presence of a protein at least as late as the LUCA could indicate a functional conservation from a much early stage, especially if there is a plausible prebiotic precursor that could have performed an analogous function.

**Fig. 2.**
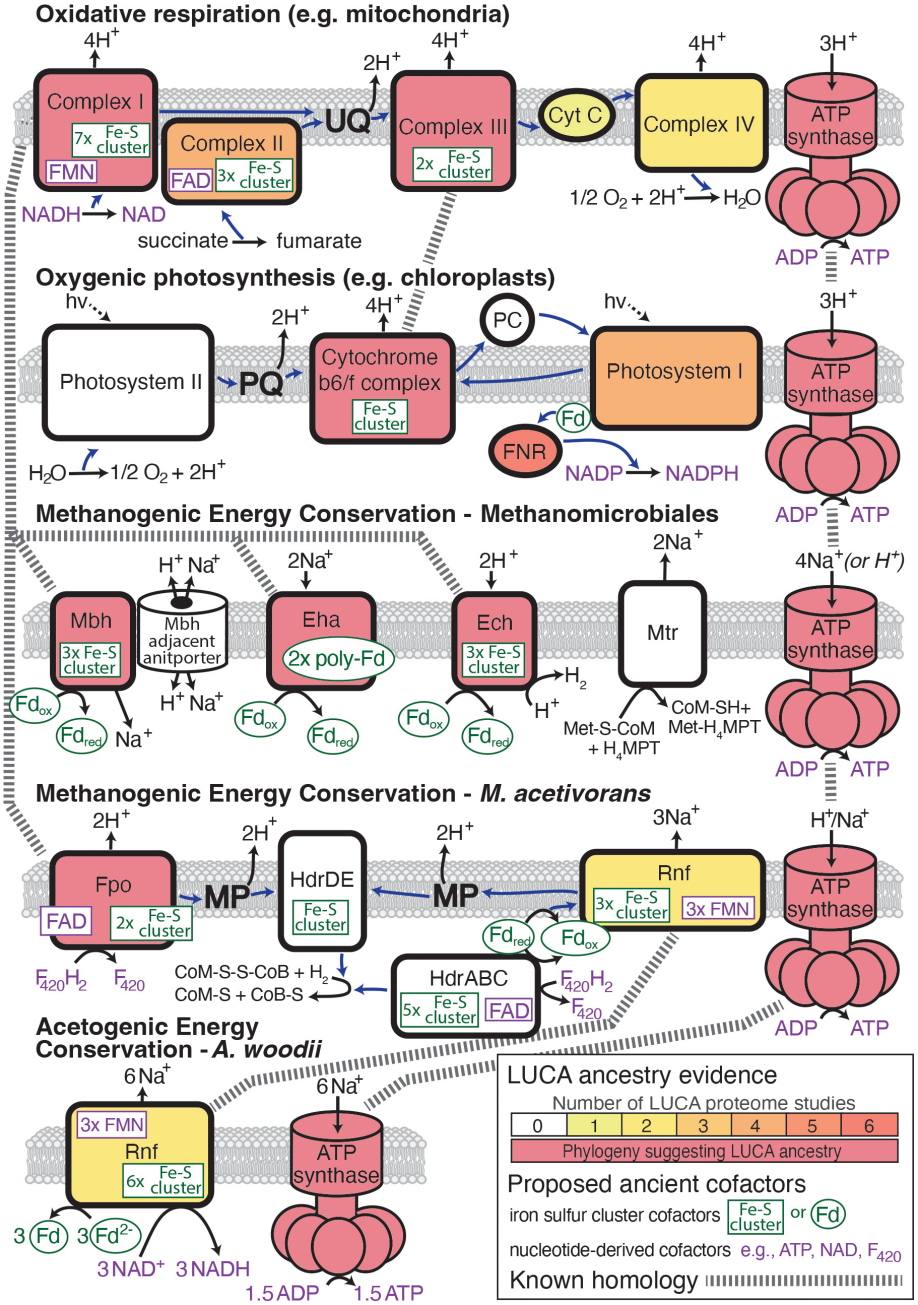
Signatures of early evolution across different types of chemiosmotic energy conservation. Electron flow is shown as blue arrows. Likely ancestry from the LUCA is reflected by either direct phylogenetic evidence or the number of different LUCA proteome studies (out of eight total) that predict a component of the complex to be descended from the LUCA (*SI Appendix*) (38, 64). Protein cofactors that are potential relics of prebiotic mineral catalysis (65) or ribozyme catalysts (66) are highlighted in green and purple, respectively. Homology across different ETC components is indicated by a dashed line. Electron carrier proteins that are components of ETC complexes such as cytochrome B are not shown.

## Common Features Across Diverse ETCs

The first ETC that most biology students learn is that of the mitochondrion, an organelle that was inherited by an ancestral eukaryote via endosymbiosis of an ancestral alphaproteobacterium (or close relative) (67–70). In mitochondria, the ETC consists of four complexes, three of which act to pump protons across a membrane. Electrons are inputted by NADH via Complex I, or FADH_2_ via Complex II, and the final electron acceptor is dissolved oxygen, which is reduced to form H_2_O (Fig. 2). The organic compound, ubiquinone, and the small protein, Cytochrome C, carry electrons between complexes. Ubiquinone itself contributes to the proton gradient as it is reduced near one side of the membrane and oxidized near the other, thereby moving protons across the membrane as it cycles between redox states.

In the bacterial domain, a range of anaerobic respiration ETCs make use of terminal electron acceptors other than oxygen (71, 72). Common forms of anaerobic respiration include sulfate reduction (SO_4_^2−^ to H_2_S or elemental S), iron oxide reduction [Fe(III) to Fe(II)], dissimilatory nitrate reduction to ammonium or DNRA (NO_3_^−^ to NH_4_^+^), manganese oxide reduction [Mn(IV) to Mn(II)], and denitrification (NO_3_^−^ to N_2_). The different forms of respiration require different ETC components, but even so, many features are conserved. For example, these anaerobic respiratory chains often use similar mobile electron carriers such as cytochrome C, a quinone (often menaquinone rather than ubiquinone), and homologs of one or more of the membrane-bound proton pumping complexes found in aerobic respiration.

Beyond respiration, ETCs are also used for energy conservation in photosynthesis. The canonical chloroplast ETC found in plants includes two photosystems that absorb photons and, in doing so, transfer electrons at high redox potential energy (Fig. 2) (73, 74). The initial source of electrons comes from splitting water to molecular oxygen and protons, the latter contributing to the proton gradient. These electrons are used to reduce a quinone, plastoquinone, which, like ubiquinone, can shuttle protons across the membrane as it is oxidized and reduced. Plastoquinone then reduces the sole proton pumping complex, cytochrome b6f, a homolog of Complex III from aerobic respiration, which then reduces a small protein, plastocyanin. The plastocyanin then passes the electron to a second photosystem, which excites the electron via photon absorption. This second photosystem (confusingly called Photosystem I) can then either pass the excited electron back to plastoquinone to pump more protons through the cytochrome b6f complex or to a series of proteins, ferredoxin and ferredoxin nicotinamide adenine dinucleotide phosphate (NADP) reductase (FNR), which ultimately reduce NADP to NADPH. The redox potential energy of NADPH is then used for carbon fixation.

Several forms of anoxygenic photosynthesis exist within bacteria and other forms of heterotrophic phototrophy are used by both bacteria and archaea. The simplest form of phototrophy is found in haloarchaea, in which the membrane protein, bacteriorhodopsin, uses light absorption to directly pump protons across the membrane (75, 76). This form of single-protein phototrophy can produce ATP through the proton gradient it generates, but does not capture redox potential energy and does not have an associated ETC. A related bacteriorhodopsin has been shown to pump sodium, although it is not known whether the resulting sodium gradient is used for ATP generation (77). Other bacterial forms of anoxygenic photosynthesis such as those found in purple bacteria and green sulfur bacteria have ETCs powered by a single photosystem each. The photosystems of the purple sulfur bacteria and green sulfur bacteria are distinct from one another, but appear to be related to oxygenic photosystem II and photosystem I, respectively (78). These anoxygenic photosynthetic ETCs also use quinones (79) and homologs of Complex III from aerobic respiration (80).

Chemoautotrophy represents yet another metabolic strategy, distinct from both phototrophy and respiration. There are many different forms of chemoautotrophy in both bacteria and archaea, including methanogenesis, anaerobic ammonium oxidation (anammox), nitrification, sulfur oxidation and reduction, and hydrogen oxidation. The energy conserving ETCs associated with different chemoautotrophic metabolisms are likewise distinct from one another (81–84) and not all of them have been well described. Even within methanogenesis, one of the most wide-spread forms of chemoautotrophic metabolism, there are several distinct membrane bioenergetic systems that share only the Mtr complex between them (85, 86). While a thorough account of the different forms of methanogenesis and their associated membrane bioenergetic systems is beyond the scope of this article, two different examples are represented in Fig. 2 (and for a more detailed review, see 86 and 87).

The first example shown in Fig. 2 is derived from a recent pangenome study of the order Methanomicrobiales (88). This energy conservation system is linked to CO_2_-reducing hydrogenotrophic methanogenesis, possibly the earliest form of methanogenesis (87). It contains several hydrogenases that are homologous to Complex I in the aerobic respiration ETC (89–92). These hydrogenases, however, do not transfer electrons to one another and thus do not form an ETC. Rather, they convert chemiosomotic energy from the ion/proton gradient into reduced ferredoxin that is used in methanogenesis and biosynthetic metabolism. The conserved MTR complex acts to establish a sodium ion gradient across the membrane and Na^+^/H^+^ antiporters potentially enable some Methanomicrobiales species to use H^+^ and Na^+^ gradients concurrently for ATP synthesis (88).

A second example shown in Fig. 2 is from *Methanosarcina acetivorans* (93). Here, acetotrophic methanogenesis is connected to an ETC composed of two different systems that both receive electrons from reduced F_420_H_2_ cofactors. A methanophenazine electron carrier accepts electrons from both systems and moves protons across a membrane via oxidation and reduction cycles in a manner similar to the quinones in respiratory and phototrophic ETCs. The only proton pump complex, F_420_H_2_ dehydrogenase (Fpo), is found in one of these two ETC systems. This proton pump has subunits that are evolutionarily related to subunits of Complex I in the aerobic respiration ETC (89). The system also includes an ion pump, Rnf, that pumps sodium ions across the membrane, which can be used along with protons to power the ATP synthase motor complex. Despite containing many components that are homologous to the ETCs of other organisms, this ETC likely evolved relatively recently (87).

This Rnf complex is also found in the bacterium *Acetobacterium woodii*, which performs homoacetogenic fermentation (Fig. 2) (94). Here, the Rnf complex also pumps sodium ions across the membrane which are used to generate ATP, but Rnf is the only energy conserving membrane complex other than the ATP synthase, so there is no ETC (95). Anammox bacteria have a partially described ETC that may contain homologues of the Complexes I and III from the aerobic respiration ETC as well as menaquinone and a putative cytochrome C homolog (82, 96). The ETC of the hydrogen oxidizing and sulfur reducing bacterium, *Aquifex aeolicus,* is only partially described, but also appears to contain homologs of Complexes I and III and uses quinones, possibly ubiquinones, as electron carriers (83).

While proton/ion pumping is perhaps the most common mechanism for generating a proton or ion motive force, several other mechanisms can contribute to these gradients. A second mechanism of generating a proton gradient is via a redox loop. Protons can be moved across the membrane when an electron carrier, for example, a quinone, is reduced while facing one side of the membrane, thereby also binding protons as hydrogens, and then oxidized while facing the other side of the membrane, thereby releasing those hydrogens as protons (97). Proton gradients can also be enhanced by substrate turnover, wherein protons are consumed on one side and/or produced on the other side of the membrane. This phenomenon is observed in oxygenic photosynthesis, where on one side of the membrane, protons are produced when water molecules are split, while on the other side of the membrane, protons are consumed as NADPH is formed from NADP^+^. Another example of this phenomenon is found in *Pyrococcus* *furiosus* (98) which has a respiratory system consisting of a single hydrogenase enzyme, MBH, that takes electrons from reduced ferredoxin and uses them to form H_2_ from two protons.

Taken together, there is a very broad range of metabolic strategies observed across the bacteria and archaea, and consequently a diverse array of energy conserving ETCs. However, there are similarities and evolutionary relationships across very different types of ETCs (79, 80, 89, 99, 100). In addition to the ATP synthase complex that is universal across all chemiosmotic energy conservation systems, homologs of Complexes I and III from the aerobic respiration ETC are found in ETCs associated with anaerobic respiration, anoxygenic and oxygenic photosynthesis, methanogenesis, anammox, and hydrogen oxidation. Quinones act as mobile electron carriers in nearly all ETCs aside from those in methanogens. Also, within these conserved complexes are common metal cofactors and organic nucleotide-derived cofactors such as iron sulfur clusters, NADH, and FADH_2_, which have been proposed to represent catalysts from earlier stages of metabolic evolution (101). Despite the apparent diversity of ETCs across the tree of life, these commonalities and evolutionary relationships suggest that at least some components may have been mutually inherited from ancient common ancestors.

## The Early Evolution of Membrane Bioenergetics

**Fig. 3.**
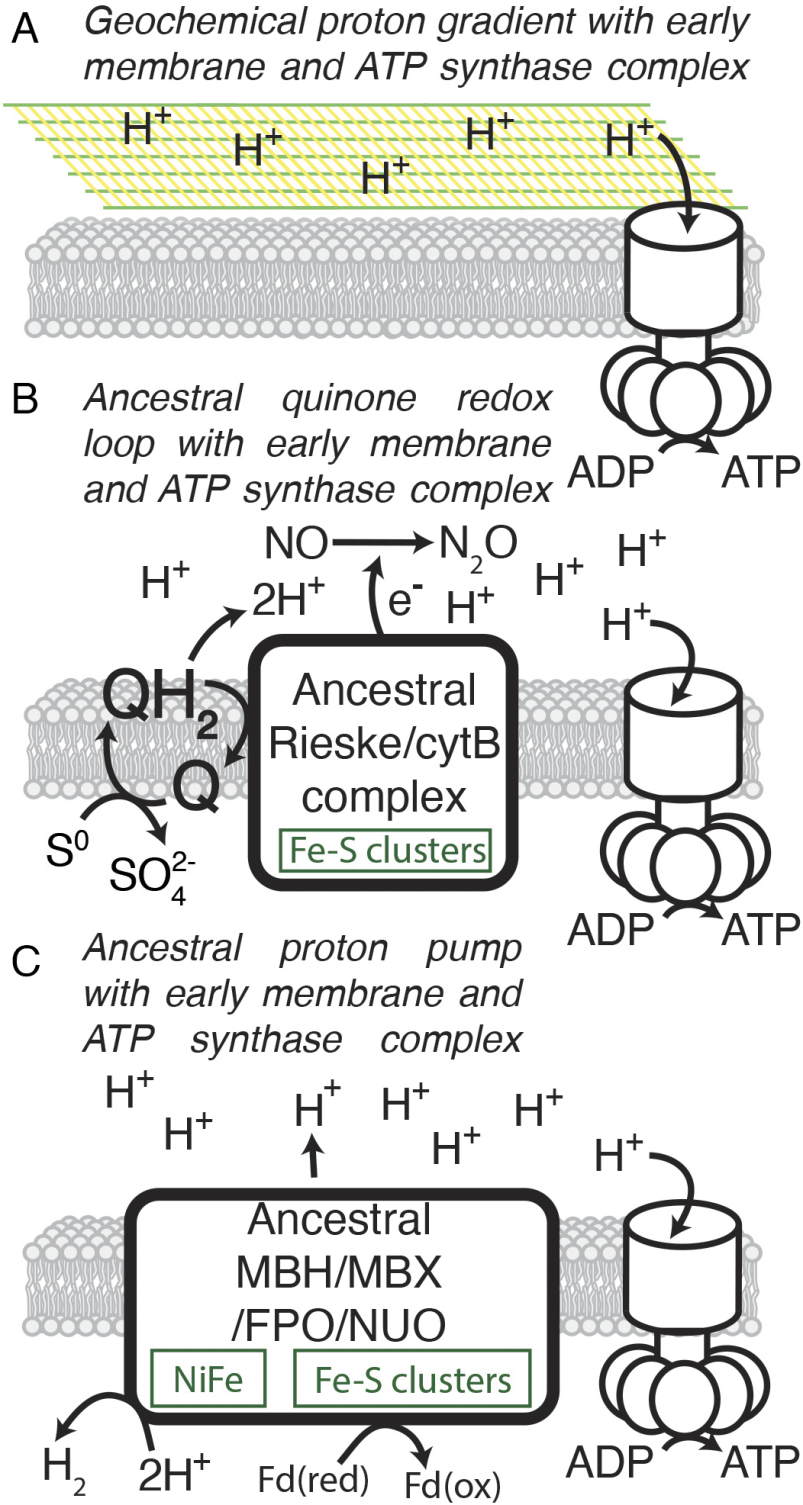
Previously published hypotheses of ancient chemiosmotic energy conservation systems based on phylogenetic evidence. (*A*) An ancestral ATP synthase motor complex is powered by a geochemical proton gradient produced in an alkaline vent environment (103, 109). (*B*) An ancestor of Rieske/cytB proteins (Complex III) produces a proton gradient through a quinone-based redox loop (100) in which quinones and the ancestor of Rieske/cytB complex link the oxidation of S^0^ and the reduction of nitric oxide (110). (*C*) An ancestor to the MBH, MBX, FPO, and Nuo (Complex I) complexes produces a proton gradient through proton pumping activity and substrate turnover (89). Phylogenetic evidence suggests that ancestors of all three of these ETC components were present at the time of the LUCA.

Phylogenetic evidence strongly suggests that the ATP synthase complex predated the LUCA because the catalytic and noncatalytic subunits of ATP synthase are members of the same family of universal paralogs and were likely both present in the proteome of the LUCA (57, 58, 60). This very early origin of the ATP synthase complex has led to a model proposing that, before ETCs, ATP generation could have been powered by geochemical proton gradients (102) produced by serpentinite-hosted alkaline hydrothermal vents (Fig. 3) (28, 103, 104) in some ways analogous to the Lost City Hydrothermal Field, which produces an alkaline vent fluid as serpentine is formed through the hydration of olivine-bearing ocean crust (26, 105). In an early Earth context, the interface between alkaline hydrothermal vent fluid and mildly acidic early Earth seawater (106, 107) could have generated a proton gradient that powered an ancient ATP synthase (103, 108).

Despite the clear phylogenetic evidence of an ancient ATP synthase motor complex, the role of cellular membranes in early evolution has been controversial (42). Under certain conditions, membranes readily form from abiotic amphiphilic compounds (16, 111) and from prebiotically plausible compounds under simulated marine alkaline vent conditions (112). Despite this propensity for prebiotic membranes to form in early Earth settings, biological evidence from extant organisms is often interpreted as undermining an early evolution of cellular organization. Archaeal phospholipids are composed of branched fatty acids linked to a L-glycerol by an ether bond, while bacterial phospholipids are composed of unbranched fatty acids linked to a D-glycerol by an ester bond. Some have argued that this difference suggests that early life, even by the time of the LUCA, was noncellular (113) or semicellular (27). This assumed semicellularity has led others to explain the pre-LUCA origin of the ATP synthase by suggesting that it had originally served a different function, as a translocation channel for nucleic acids or proteins (114).

But this difference between archaeal and bacterial phospholipids need not preclude the presence of cellular membranes in early evolutionary history. Early organisms may have had a mixed phospholipid membrane (42) or one of the phospholipid types may have evolved from the other following the divergence of the bacterial and archaeal lineages. Similarity between archaeal and bacterial phospholipid biosynthesis pathways supports this view (42). Indeed, phylogenetic evidence shows that the machinery for embedding proteins into membranes and transporting them across membranes emerged prior to the LUCA (41), suggesting that membranes were present prior to the LUCA despite the ambiguity regarding their composition. Given the evidence that cellular membranes were present prior to the LUCA, the proposal that the ATP synthase complex served some other function in early life seems unconvincing (100), especially since a true system for membrane translocation appears to be just as ancient as the ATP synthase complex (41).

Current evidence, therefore, suggests that biological membranes and the ATP synthase motor complex evolved prior to the time of the LUCA. In addition to ATP synthase, several individual components of ETCs appear to have also evolved by the time of the LUCA (Fig. 3). Even components of ETCs that involve molecular oxygen and thus were unlikely to have been present in their complete form until well after the time of the LUCA (115) appear to have homologs that date to the LUCA if not earlier (99). Both protein structure comparisons and protein sequence phylogenies show that the ancestors of several ETC complex subunits were likely present in the proteome of the LUCA (89, 100, 116). These include both the Rieske protein subunit and cytochrome B, which are found together in Complex III of aerobic respiration, the cytochrome b6f complex of oxygenic photosynthesis, and others (117); molybdopterin subunits of DMSO reductases, which are found in ETCs associated with multiple types of anaerobic respiration; and the [NiFe] hydrogenases, which are found in Complex I of aerobic respiration and several related complexes found in bacteria and archaea (Fig. 2) (89–91).

While phylogenetic analysis shows with high certainty that ancestors of these protein families were present in the proteome of the LUCA, their ancestral functions can still be ambiguous. The observation that ancestral Rieske proteins and cytochrome B were present in the proteome of the LUCA may indicate that they contributed to a proton gradient through proton pumping activity, a quinone-based redox loop, or both (100), possibly using S^0^ as an electron donor and NO as an electron acceptor (110). Similarly, the observation that an ancestral [NiFe] hydrogenase was present in the LUCA does not, on its own, elucidate the ancestral function (89). Extant homologs of this protein are found in Complex I or NADH quinone oxidoreductase (NUO) in the aerobic respiration ETC, the FPO complex found in the ETCs of some methanogens, the Na^+^/H^+^ transporting MBH complex, and the MBX complex associated with elemental sulfur reduction. Schut et al. (89) infer that the ancestor of NUO, FPO, MBH, and MBX, had a function most similar to MBH because the redox environment in which MBH functions is most similar to the likely redox environment of the LUCA. In all of these cases, the ambiguity of ancestral function could be resolved by future research through the combination of ancestral sequence reconstruction, i.e., inferring the sequence of the LUCA-associated ancestral protein, followed by protein structure and function prediction on that inferred ancestral sequence.

## Reconstructing Prebiotic ETCs and Proton Gradients

Various authors have proposed that some components of chemiosmosis, including aspects of ETC enzymes, proton gradient generation, and formation of ATP or polyphosphate energy currency, could have functioned in a prebiotic setting at or prior to the origin of life (26, 103). Though a complete prebiotic pathway similar to a biological ETC, involving discrete reactions that can be individually tested, has not yet been demonstrated in the lab, some aspects of ETC systems have been shown to function in a prebiotic context. Recent experiments have demonstrated that the ancient cofactors found across diverse ETCs can perform similar reactions even in the absence of their modern protein complexes.

For example, NAD^+^ can be nonenzymatically reduced by pyruvate (118), somewhat analogous to the reduction of NAD^+^ by pyruvate decarboxylation during aerobic respiration. FeS minerals can drive the reduction of NAD^+^ to NADH under early Earth conditions, which is analogous to the electron transfer from NADH to the Fe-S clusters in Complex I (119, 120). NAD^+^ reduction has been linked to membrane electron transfer via a photocatalytically generated pH gradient and associated electron transfer through a protocell membrane, though involving some prebiotically implausible components (121). It has also been shown that prebiotic Fe-S peptides or metal-porphyrin complexes can promote redox reactions involving NAD^+^/NADH, and abiotically transfer those electrons to and from a quinone (122, 123). These different reactions were conducted with variations in what is considered prebiotic conditions (124, 125), and at different simulated stages in chemical evolution (for example, a geological FeS mineral vs. an FeS peptide). While the prebiotic feasibility of ETC-like geochemistry has only recently been addressed experimentally, these studies demonstrate the importance of geochemical conditions in the evolution of the first ETCs.

Another component of ETCs is the proton gradients that they generate. Recent experiments have produced proton gradients across protocell membranes through potential prebiotic mechanisms. For example, redox transformations of NAD^+^/NADH have been accomplished inside a protocell in a manner that generates a pH gradient, either via generation of OH^-^ from H_2_O_2_ reduction inside the membrane (122) or via H^+^ generation by reaction of a photocatalytic mineral outside the membrane (121). In laboratory experiments simulating prebiotic hydrothermal chimneys (or chemical gardens), the gradients between interior and exterior solutions generate measurable potentials across the mineral precipitate walls (126, 127). The gradients generated in hydrothermal chimney or chemical garden experiments are a combination of ΔpH and ion gradients, maintained by the influx of the interior solution and in equilibrium with the precipitation of minerals and ion diffusion through the mineral membrane (128). Redox gradients in prebiotic hydrothermal systems may also be possible in the presence of electron donors (e.g., hydrothermally produced H_2_ and CH_4_) (129) and acceptors (e.g., seawater CO_2_) (106) in a vent system, and some electrochemically active mineral phases could act as geo-electrodes driving prebiotic redox reactions of these species (130, 131).

Analogs of ATP/polyphosphate synthesis have also been proposed and studied in prebiotic and geochemical systems (132–135). These are typically condensation or substrate phosphorylation reactions driven by heat or mineral surface reactions (133, 134, 136, 137) generating pyrophosphate or ATP in equilibrium with their hydrolysis products, which is not mechanistically similar to the proton gradient–driven ATP synthase enzyme that generates ATP at a very high disequilibrium within cells. Some hypotheses for a precursor to ATP synthase have been proposed, including proton gradients acting across a mineral membrane containing the iron hydroxide mineral green rust (138, 139); or the prebiotic peptide nesting of phosphate leading to the emergence of a proton-translocating pyrophosphatase (132, 140–142). While one recent study demonstrated that pyrophosphate can form through substrate-level phosphorylation in a system with a pH gradient across a microfluidic iron hydroxide membrane (137), no published experiment has shown a geochemical mechanistic analog to ATP synthase.

Many of these prebiotic and geochemical analogs to different components of ETCs have only recently been published. Despite the nascence of this research topic, experiments have demonstrated in principle that some parts of the electron transfer chemistry, proton gradient generation, and phosphate bond formation, foundational to all modern ETCs, can be replicated in the lab without protein components and under geochemically plausible conditions. Demonstrating the prebiotic plausibility of an abiotic, ETC-like system does not on its own indicate that such a scenario was present during the origin of life. But this research paired with a deeper understanding of the very early evolutionary history of ETCs from phylogenetic analysis could shed light on how the first ETCs evolved, when they evolved with respect to the origin of life, and what geochemical environments facilitated that evolutionary process.

## Understanding the Origin of Membrane Bioenergetics through Bottom–Up and Top–Down Research

The complex cellular organisms depicted by LUCA proteome reconstructions appear to have had both a genetic system and cellular organization similar to that of many extant prokaryotes (Fig. 1). Membrane bioenergetics requires both a translation system to produce protein complexes and a fluid mosaic membrane to maintain a chemiosmotic gradient. Membrane bioenergetics, therefore, would have been possible prior to the time of the LUCA, but only after both of these systems had evolved. The ATP synthase complex remains the only ETC component belonging to a family of universal paralogs and thus has the strongest phylogenetic support for ancestry dating to before the time of the LUCA. However, the observation that more than one ETC component can be shown through phylogenetic analysis to have been present at least as late as the time of the LUCA suggests that chemiosmotic energy conservation in general first evolved even earlier.

While phylogenetic inference may not be able to access these earliest stages of pre-LUCA evolution, bottom-up research can provide prebiotic evidence that, together with our current understanding of early ETC components, depicts possible scenarios in which these ancient ETC components replaced even earlier, pre-protein analogs. Geochemistry and simple organic compounds may have facilitated electron transfer (118, 120), generation and maintenance of a proton gradient (121, 122), and even pyrophosphate/nucleotide polyphosphate formation (133, 137). The semienzymatic hypothesis states that metabolic pathways may have first emerged because genetically-encoded enzymes evolved to replace prebiotic catalysts (143). This hypothesis has guided protometabolic research (133, 137). A similar approach should be adopted to understand the origin of membrane bioenergetics (144, 145).

Several precursor chemiosmotic energy conservation systems proposed by previous authors to have been present at or prior to the time of the LUCA are depicted in Fig. 3 (89, 100, 103, 109). Experiments simulating prebiotic geochemistry and protocells may test the feasibility of preprotein precursors to these early energy conservation strategies. For example, simulated alkaline vent mineral precipitates have been shown to drive pyrophosphate formation through substrate level phosphorylation (133, 137), but it has yet to be shown that these systems produce a proton gradient strong enough to power chemiosmotic ATP synthesis (Fig. 3*A*). Similarly, the abiotic synthesis of a quinone from prebiotically available precursors was recently demonstrated (146). Protocell experiments could reveal whether a quinone-based redox loop on prebiotically plausible redox pairs could establish a proton gradient (Fig. 3*B*). Protocell experiments could also demonstrate whether proton gradients could be produced through substrate turnover as in the formation of diatomic hydrogen from proton reduction catalyzed by a prebiotic precursor of the inorganic cofactors found in ETC complexes (Fig. 3*C*).

Taken together, certain components of ETCs appear to be ancient, some dating back to a time prior to the LUCA. At the same time, recent laboratory research on prebiotic chemistry and protocells has advanced to the point where some steps in ETCs can be performed in simulated early Earth environments in the absence of proteins. Still, other key steps in a prebiotic ETC remain experimentally unexamined. By pairing these top down and bottom up research approaches and advancing our understanding using both phylogenetic and laboratory techniques, future research may uncover a detailed account of the earliest evolution of membrane bioenergetics, perhaps deepening our understanding of the origin of life itself.

## Supplementary Material

Appendix 01 (PDF)

## Data Availability

All study data are included in the article and/or *SI Appendix*.

## References

[1] A. C. Allwood, M. R. Walter, B. S. Kamber, C. P. Marshall, I. W. Burch, Stromatolite reef from the Early Archaean era of Australia. Nature **441**, 714–718 (2006). 16760969 10.1038/nature04764

[2] E. A. Bell, P. Boehnke, T. M. Harrison, W. L. Mao, Potentially biogenic carbon preserved in a 4.1 billion-year-old zircon. Proc. Natl. Acad. Sci. U.S.A. **112**, 14518–14521 (2015). 26483481 10.1073/pnas.1517557112 PMC4664351

[3] H. C. Betts , Integrated genomic and fossil evidence illuminates life’s early evolution and eukaryote origin. Nat. Ecol. Evol. **2**, 1556–1562 (2018). 30127539 10.1038/s41559-018-0644-x PMC6152910

[4] A. A. T. Davín , Gene transfers can date the tree of life. Nat. Ecol. Evol. **2**, 904–909 (2018). 29610471 10.1038/s41559-018-0525-3 PMC5912509

[5] J. M. Wolfe, G. P. Fournier, Horizontal gene transfer constrains the timing of methanogen evolution. Nat. Ecol. Evol. **2**, 897–903 (2018). 29610466 10.1038/s41559-018-0513-7

[6] S. L. Miller, A production of amino acids under possible primitive earth conditions. Science **117**, 528–529 (1953). 13056598 10.1126/science.117.3046.528

[7] H. J. Cleaves, J. H. Chalmers, A. Lazcano, S. L. Miller, J. L. Bada, A reassessment of prebiotic organic synthesis in neutral planetary atmospheres. Orig. Life Evol. Biosph. **38**, 105–115 (2008). 18204914 10.1007/s11084-007-9120-3

[8] M. Levy, S. L. Miller, K. Brinton, J. L. Bada, Prebiotic synthesis of adenine and amino acids under Europa-like conditions. Icarus **145**, 609–613 (2000). 11543508 10.1006/icar.2000.6365

[9] W. L. Marshall, Hydrothermal synthesis of amino acids. Geochim. Cosmochim. Acta **58**, 2099–2106 (1994).

[10] Y. Furukawa , Extraterrestrial ribose and other sugars in primitive meteorites. Proc. Natl. Acad. Sci. U.S.A. **116**, 24440–24445 (2019). 31740594 10.1073/pnas.1907169116 PMC6900709

[11] A. W. Schwartz, R. M. de Graaf, The prebiotic synthesis of carbohydrates: A reassessment. J. Mol. Evol. **36**, 101–106 (1993).

[12] J. Oro, Mechanism of synthesis of adenine from hydrogen cyanide under possible primitive earth conditions. Nature **191**, 1193–1194 (1961). 13731264 10.1038/1911193a0

[13] A. C. Rios, Y. Tor, On the origin of the canonical nucleobases: An assessment of selection pressures across chemical and early biological evolution. Isr. J. Chem. **53**, 469–483 (2013). 25284884 10.1002/ijch.201300009 PMC4181368

[14] M. W. Powner, B. Gerland, J. D. Sutherland, Synthesis of activated pyrimidine ribonucleotides in prebiotically plausible conditions. Nature **459**, 239–242 (2009). 19444213 10.1038/nature08013

[15] M. Yadav, R. Kumar, R. Krishnamurthy, Chemistry of abiotic nucleotide synthesis. Chem. Rev. **120**, 4766–4805 (2020). 31916751 10.1021/acs.chemrev.9b00546

[16] D. W. P. Deamer, Amphiphilic components of the Murchison carbonaceous chondrite: Surface properties and membrane formation. Orig. Life Evol. Biosph. **19**, 21–38 (1989). 2748144 10.1007/BF01808285

[17] D. Fayolle , Crude phosphorylation mixtures containing racemic lipid amphiphiles self-assemble to give stable primitive compartments. Sci. Rep. **7**, 18106 (2017). 29273739 10.1038/s41598-017-18053-y PMC5741756

[18] K. Chandru , Simple prebiotic synthesis of high diversity dynamic combinatorial polyester libraries. Commun. Chem. **1**, 30 (2018).

[19] J. G. Forsythe , Surveying the sequence diversity of model prebiotic peptides by mass spectrometry. Proc. Natl. Acad. Sci. U.S.A. **114**, E7652–E7659 (2017). 28847940 10.1073/pnas.1711631114 PMC5604043

[20] J. G. Forsythe , Ester-mediated amide bond formation driven by wet-dry cycles: A possible path to polypeptides on the prebiotic Earth. Angew. Chem. Int. Ed. Engl. **54**, 9871–9875 (2015). 26201989 10.1002/anie.201503792 PMC4678426

[21] B. A. Anderson , The unexpected base-pairing behavior of cyanuric acid in RNA and ribose versus cyanuric acid induced helicene assembly of nucleic acids: Implications for the Pre-RNA paradigm. Chemistry **27**, 4033–4042 (2021). 33174270 10.1002/chem.202004397

[22] B. J. Cafferty, D. M. Fialho, J. Khanam, R. Krishnamurthy, N. V. Hud, Spontaneous formation and base pairing of plausible prebiotic nucleotides in water. Nat. Commun. **7**, 11328 (2016). 27108699 10.1038/ncomms11328 PMC4848480

[23] D. M. Fialho , Depsipeptide nucleic acids: Prebiotic formation, oligomerization, and self-assembly of a new proto-nucleic acid candidate. J. Am. Chem. Soc. **143**, 13525–13537 (2021). 34398608 10.1021/jacs.1c02287

[24] J. A. Baross, S. E. Hoffman, Submarine hydrothermal vents and associated gradient environments as sites for the origin and evolution of life. Orig. Life Evol. Biosphere **15**, 327–345 (1985).

[25] B. Damer, D. Deamer, The hot spring hypothesis for an origin of life. Astrobiology **20**, 429–452 (2020). 31841362 10.1089/ast.2019.2045 PMC7133448

[26] W. Martin, J. Baross, D. Kelley, M. J. Russell, Hydrothermal vents and the origin of life. Nat. Rev. Microbiol. **6**, 805–814 (2008). 18820700 10.1038/nrmicro1991

[27] W. Martin, M. J. Russell, On the origins of cells: A hypothesis for the evolutionary transitions from abiotic geochemistry to chemoautotrophic prokaryotes, and from prokaryotes to nucleated cells. Philos. Trans. R. Soc. Lond. B Biol. Sci. **358**, 59–83; discussion 83–55 (2003). 12594918 10.1098/rstb.2002.1183 PMC1693102

[28] M. J. Russell, A. J. Hall, "The onset and early evolution of life" in Evolution of Early Earth’s Atmosphere, Hydrosphere, and Biosphere—Constraints from Ore Deposits, S. E. Kesler, H. Ohmoto, Eds. (Geological Society of America, Boulder, CO, 2006), pp. 1–32.

[29] G. D. Cody , Primordial carbonylated iron-sulfur compounds and the synthesis of pyruvate. Science **289**, 1337–1340 (2000). 10958777 10.1126/science.289.5483.1337

[30] G. Wachtershauser, Before enzymes and templates: Theory of surface metabolism. Microbiol. Rev. **52**, 452–484 (1988). 3070320 10.1128/mr.52.4.452-484.1988 PMC373159

[31] J. P. Ferris, G. Ertem, Montmorillonite catalysis of RNA oligomer formation in aqueous solution. A model for the prebiotic formation of RNA. J. Am. Chem. Soc. **115**, 12270–12275 (1993). 11540110 10.1021/ja00079a006

[32] H. Hartman, Speculations on the origin and evolution of metabolism. J. Mol. Evol. **4**, 359–370 (1975). 1206724 10.1007/BF01732537

[33] H. Trinks, W. Schroder, C. K. Biebricher, Ice and the origin of life. Orig. Life Evol. Biosphs. **35**, 429–445 (2005). 10.1007/s11084-005-5009-1 16231207

[34] E. E. A. Stüeken , Did life originate from a global chemical reactor? Geobiology **11**, 101–126 (2013). 23331348 10.1111/gbi.12025

[35] A. P. Pross, The origin of life: What we know, what we can know and what we will never know. Open Biol. **3**, 120190 (2013). 23466673 10.1098/rsob.120190 PMC3718341

[36] A. Becerra, L. Delaye, S. Islas, A. Lazcano, The very early stages of biological evolution and the nature of the last common ancestor of the three major cell domains. Annu. Rev. Ecol. Evol. Syst. **38**, 361–379 (2007).

[37] S. J. Berkemer, S. E. McGlynn, A new analysis of archaea-bacteria domain separation: Variable phylogenetic distance and the tempo of early evolution. Mol. Biol. Evol. **37**, 2332–2340 (2020). 32316034 10.1093/molbev/msaa089 PMC7403611

[38] A. J. Crapitto, A. Campbell, A.-J. Harris, A. D. Goldman, A consensus view of the proteome of the last universal common ancestor. Ecol. Evol. **12**, e8930 (2022). 35784055 10.1002/ece3.8930 PMC9165204

[39] G. Caetano-Anolles , The origin and evolution of modern metabolism. Int. J. Biochem. Cell Biol. **41**, 285–297 (2009). 18790074 10.1016/j.biocel.2008.08.022

[40] A. D. Goldman, J. T. Beatty, L. F. Landweber, The TIM barrel architecture facilitated the early evolution of protein-mediated metabolism. J. Mol. Evol. **82**, 17–26 (2016). 26733481 10.1007/s00239-015-9722-8 PMC4709378

[41] A.-J. Harris, A. D. Goldman, The very early evolution of protein translocation across membranes. PLoS Comput. Biol. **17**, e1008623 (2021). 33684113 10.1371/journal.pcbi.1008623 PMC7987157

[42] J. Pereto, P. Lopez-Garcia, D. Moreira, Ancestral lipid biosynthesis and early membrane evolution. Trends Biochem. Sci. **29**, 469–477 (2004). 15337120 10.1016/j.tibs.2004.07.002

[43] A. D. Goldman, L. F. Landweber, Oxytricha as a modern analog of ancient genome evolution. Trends Genet. **28**, 382–388 (2012). 22622227 10.1016/j.tig.2012.03.010 PMC3401270

[44] G. M. Nagel, R. F. Doolittle, Phylogenetic analysis of the aminoacyl-tRNA synthetases. J. Mol. Evol. **40**, 487–498 (1995). 7783224 10.1007/BF00166617

[45] A. M. Poole , The case for an early biological origin of DNA. J. Mol. Evol. **79**, 204–212 (2014). 25425102 10.1007/s00239-014-9656-6 PMC4247479

[46] M. P. G. Hoeppner, P. P. Poole, Comparative analysis of RNA families reveals distinct repertoires for each domain of life. PLoS Comput. Biol. **8**, e1002752 (2012). 23133357 10.1371/journal.pcbi.1002752 PMC3486863

[47] E. Fer, K. M. McGrath, L. Guy, A. J. Hockenberry, B. Kacar, Early divergence of translation initiation and elongation factors. Protein Sci. **31**, e4393 (2022). 36250475 10.1002/pro.4393 PMC9601768

[48] G. P. Fournier, E. J. Alm, Ancestral reconstruction of a Pre-LUCA aminoacyl-tRNA synthetase ancestor supports the late addition of trp to the genetic code. J. Mol. Evol. **80**, 171–185 (2015). 25791872 10.1007/s00239-015-9672-1

[49] G. P. Fournier, C. P. Andam, E. J. Alm, J. P. Gogarten, Molecular evolution of aminoacyl tRNA synthetase proteins in the early history of life. Orig. Life Evol. Biosphs. **41**, 621–632 (2011). 10.1007/s11084-011-9261-2 22200905

[50] A. Mushegian, Gene content of LUCA, the last universal common ancestor. Front. Biosci. **13**, 4657–4666 (2008). 18508537 10.2741/3031

[51] A. S. Petrov , History of the ribosome and the origin of translation. Proc. Natl. Acad. Sci. U.S.A. **112**, 15396–15401 (2015). 26621738 10.1073/pnas.1509761112 PMC4687566

[52] C. Woese, The universal ancestor. Proc. Natl. Acad. Sci. U.S.A. **95**, 6854–6859 (1998). 9618502 10.1073/pnas.95.12.6854 PMC22660

[53] F. H. Crick, The origin of the genetic code. J. Mol. Biol. **38**, 367–379 (1968). 4887876 10.1016/0022-2836(68)90392-6

[54] R. D. Knight, S. J. Freeland, L. F. Landweber, Selection, history and chemistry: The three faces of the genetic code. Trends Biochem. Sci. **24**, 241–247 (1999). 10366854 10.1016/s0968-0004(99)01392-4

[55] E. N. Trifonov, Consensus temporal order of amino acids and evolution of the triplet code. Gene **261**, 139–151 (2000). 11164045 10.1016/s0378-1119(00)00476-5

[56] C. R. Woese, On the evolution of the genetic code. Proc. Natl. Acad. Sci. U.S.A. **54**, 1546–1552 (1965). 5218910 10.1073/pnas.54.6.1546 PMC300511

[57] J. M. Kollman, R. F. Doolittle, Determining the relative rates of change for prokaryotic and eukaryotic proteins with anciently duplicated paralogs. J. Mol. Evol. **51**, 173–181 (2000). 10948274 10.1007/s002390010078

[58] O. Zhaxybayeva, P. Lapierre, J. P. Gogarten, Ancient gene duplications and the root(s) of the tree of life. Protoplasma **227**, 53–64 (2005). 16389494 10.1007/s00709-005-0135-1

[59] S. Gribaldo, P. Cammarano, The root of the universal tree of life inferred from anciently duplicated genes encoding components of the protein-targeting machinery. J. Mol. Evol. **47**, 508–516 (1998). 9797401 10.1007/pl00006407

[60] J. P. Gogarten, L. Taiz, Evolution of proton pumping ATPases: Rooting the tree of life. Photosynth. Res. **33**, 137–146 (1992). 24408574 10.1007/BF00039176

[61] P. Mitchell, Coupling of phosphorylation to electron and hydrogen transfer by a chemi-osmotic type of mechanism. Nature **191**, 144–148 (1961). 13771349 10.1038/191144a0

[62] V. Muller, G. Gruber, ATP synthases: Structure, function and evolution of unique energy converters. Cell Mol. Life Sci. **60**, 474–494 (2003). 12737308 10.1007/s000180300040 PMC11138706

[63] K. Schlegel, V. Leone, J. D. Faraldo-Gomez, V. Muller, Promiscuous archaeal ATP synthase concurrently coupled to Na+ and H+ translocation Proc. Natl. Acad. Sci. U.S.A. **109**, 947–952 (2012). 22219361 10.1073/pnas.1115796109 PMC3271924

[64] A. D. Goldman, T. M. Bernhard, E. Dolzhenko, L. F. Landweber, LUCApedia: A database for the study of ancient life. Nucleic Acids Res. **41**, D1079–D1082 (2013). 23193296 10.1093/nar/gks1217 PMC3531223

[65] G. Wachtershauser, Groundworks for an evolutionary biochemistry: The iron-sulphur world. Prog. Biophys. Mol. Biol. **58**, 85–201 (1992). 1509092 10.1016/0079-6107(92)90022-x

[66] H. B. White, Coenzymes as fossils of an earlier metabolic state. J. Mol. Evol. **7**, 101–104 (1976). 1263263 10.1007/BF01732468

[67] M. W. Gray, Mitochondrial evolution. Cold Spring Harb. Perspect. Biol. **4**, a011403 (2012). 22952398 10.1101/cshperspect.a011403 PMC3428767

[68] L. Margulis, Origin of Eukaryotic Cells: Evidence and Research Implications for a Theory of the Origin and Evolution of Microbial, Plant, and Animal Cells on the Precambrian Earth (Yale University Press, New Haven, CT, 1970).

[69] S. A. Munoz-Gomez, J. G. Wideman, A. J. Roger, C. H. Slamovits, The origin of mitochondrial cristae from alphaproteobacteria. Mol. Biol. Evol. **34**, 943–956 (2017). 28087774 10.1093/molbev/msw298

[70] Z. Wang, M. Wu, An integrated phylogenomic approach toward pinpointing the origin of mitochondria. Sci. Rep. **5**, 7949 (2015). 25609566 10.1038/srep07949 PMC4302308

[71] H. Dang, N. Jiao, Perspectives on the microbial carbon pump with special reference to microbial respiration and ecosystem efficiency in large estuarine systems. Biogeosciences **11**, 3887–3898 (2014).

[72] K. H. Nealson, D. Saffarini, Iron and manganese in anaerobic respiration: Environmental significance, physiology, and regulation. Annu. Rev. Microbiol. **48**, 311–343 (1994). 7826009 10.1146/annurev.mi.48.100194.001523

[73] L. N. Duysens, J. Amesz, B. M. Kamp, Two photochemical systems in photosynthesis. Nature **190**, 510–511 (1961). 13725322 10.1038/190510a0

[74] D. Govindjee, L. O. Shevela, L. O. Björn, Evolution of the Z-scheme of photosynthesis: A perspective. Photosynth. Res. **133**, 5–15 (2017). 28160125 10.1007/s11120-016-0333-z

[75] O. Beja , Bacterial rhodopsin: Evidence for a new type of phototrophy in the sea. Science **289**, 1902–1906 (2000). 10988064 10.1126/science.289.5486.1902

[76] D. Oesterhelt, W. Stoeckenius, Functions of a new photoreceptor membrane. Proc. Natl. Acad. Sci. U.S.A. **70**, 2853–2857 (1973). 4517939 10.1073/pnas.70.10.2853 PMC427124

[77] K. Inoue , A light-driven sodium ion pump in marine bacteria. Nat. Commun. **4**, 1678 (2013). 23575682 10.1038/ncomms2689

[78] R. E. Blankenship, Early evolution of photosynthesis. Plant Physiol. **154**, 434–438 (2010). 20921158 10.1104/pp.110.161687 PMC2949000

[79] M. D. Collins, D. Jones, Distribution of isoprenoid quinone structural types in bacteria and their taxonomic implication. Microbiol. Rev. **45**, 316–354 (1981). 7022156 10.1128/mr.45.2.316-354.1981 PMC281511

[80] B. L. Trumpower, Cytochrome bc1 complexes of microorganisms. Microbiol. Rev. **54**, 101–129 (1990). 2163487 10.1128/mr.54.2.101-129.1990 PMC372766

[81] K. S. Auernik, R. M. Kelly, Identification of components of electron transport chains in the extremely thermoacidophilic crenarchaeon metallosphaera sedula through iron and sulfur compound oxidation transcriptomes. Appl. Environ. Microbiol. **74**, 7723–7732 (2008). 18931292 10.1128/AEM.01545-08 PMC2607173

[82] N. M. de Almeida , Membrane-bound electron transport systems of an anammox bacterium: A complexome analysis. Biochim. Biophys. Acta **1857**, 1694–1704 (2016). 27461995 10.1016/j.bbabio.2016.07.006

[83] M. Guiral , A membrane-bound multienzyme, hydrogen-oxidizing, and sulfur-reducing complex from the hyperthermophilic bacterium Aquifex aeolicus. J. Biol. Chem. **280**, 42004–42015 (2005). 16236714 10.1074/jbc.M508034200

[84] G. Kulkarni, D. M. Kridelbaugh, A. M. Guss, W. W. Metcalf, Hydrogen is a preferred intermediate in the energy-conserving electron transport chain of Methanosarcina barkeri. Proc. Natl. Acad. Sci. U.S.A. **106**, 15915–15920 (2009). 19805232 10.1073/pnas.0905914106 PMC2747218

[85] B. A. Berghuis , Hydrogenotrophic methanogenesis in archaeal phylum Verstraetearchaeota reveals the shared ancestry of all methanogens. Proc. Natl. Acad. Sci. U.S.A. **116**, 5037–5044 (2019). 30814220 10.1073/pnas.1815631116 PMC6421429

[86] R. K. Thauer, A. K. Kaster, H. Seedorf, W. Buckel, R. Hedderich, Methanogenic archaea: Ecologically relevant differences in energy conservation. Nat. Rev. Microbiol. **6**, 579–591 (2008). 18587410 10.1038/nrmicro1931

[87] P. S. Garcia, S. Gribaldo, G. Borrel, Diversity and evolution of methane-related pathways in archaea. Annu. Rev. Microbiol. **76**, 727–755 (2022). 35759872 10.1146/annurev-micro-041020-024935

[88] P. Browne , Genomic composition and dynamics among Methanomicrobiales predict adaptation to contrasting environments. ISME J. **11**, 87–99 (2017). 27552639 10.1038/ismej.2016.104 PMC5315469

[89] G. J. Schut , The role of geochemistry and energetics in the evolution of modern respiratory complexes from a proton-reducing ancestor. Biochim. Biophys. Acta **1857**, 958–970 (2016). 26808919 10.1016/j.bbabio.2016.01.010

[90] T. Friedrich, D. Scheide, The respiratory complex I of bacteria, archaea and eukarya and its module common with membrane-bound multisubunit hydrogenases. FEBS Lett. **479**, 1–5 (2000). 10940377 10.1016/s0014-5793(00)01867-6

[91] R. Hedderich, Energy-converting [NiFe] hydrogenases from archaea and extremophiles: Ancestors of complex I. J. Bioenerg. Biomembr. **36**, 65–75 (2004). 15168611 10.1023/b:jobb.0000019599.43969.33

[92] P. M. Vignais, A. Colbeau, Molecular biology of microbial hydrogenases. Curr. Issues Mol. Biol. **6**, 159–188 (2004). 15119826

[93] Z. Yan, J. G. Ferry, Electron bifurcation and confurcation in methanogenesis and reverse Methanogenesis. Front. Microbiol. **9**, 1322 (2018). 29973922 10.3389/fmicb.2018.01322 PMC6019823

[94] E. Biegel, S. Schmidt, J. M. Gonzalez, V. Muller, Biochemistry, evolution and physiological function of the Rnf complex, a novel ion-motive electron transport complex in prokaryotes. Cell Mol. Life Sci. **68**, 613–634 (2011). 21072677 10.1007/s00018-010-0555-8 PMC11115008

[95] A. Poehlein , An ancient pathway combining carbon dioxide fixation with the generation and utilization of a sodium ion gradient for ATP synthesis. PLoS One **7**, e33439 (2012). 22479398 10.1371/journal.pone.0033439 PMC3315566

[96] B. Kartal , How to make a living from anaerobic ammonium oxidation. FEMS Microbiol. Rev. **37**, 428–461 (2013). 23210799 10.1111/1574-6976.12014

[97] D. Richardson, G. Sawers, Structural biology. PMF through the redox loop. Science **295**, 1842–1843 (2002). 11884738 10.1126/science.1070366

[98] R. Sapra, K. Bagramyan, M. W. Adams, A simple energy-conserving system: Proton reduction coupled to proton translocation. Proc. Natl. Acad. Sci. U.S.A. **100**, 7545–7550 (2003). 12792025 10.1073/pnas.1331436100 PMC164623

[99] J. Castresana, M. Lübben, M. Saraste, D. G. Higgins, Evolution of cytochrome oxidase, an enzyme older than atmospheric oxygen. EMBO J. **13**, 2516–2525 (1994). 8013452 10.1002/j.1460-2075.1994.tb06541.x PMC395125

[100] A. L. Ducluzeau, B. Schoepp-Cothenet, F. Baymann, M. J. Russell, W. Nitschke, Free energy conversion in the LUCA: Quo vadis? Biochim. Biophys. Acta **1837**, 982–988 (2014). 24361840 10.1016/j.bbabio.2013.12.005

[101] A. D. Goldman, B. Kacar, Cofactors are remnants of life’s origin and early evolution. J. Mol. Evol. **89**, 127–133 (2021). 33547911 10.1007/s00239-020-09988-4 PMC7982383

[102] W. Martin, M. J. Russell, On the origin of biochemistry at an alkaline hydrothermal vent. Philos. Trans. R. Soc. Lond. B Biol. Sci. **358**, 59–83 (2007). 10.1098/rstb.2006.1881 PMC2442388 17255002

[103] N. Lane, J. F. Allen, W. Martin, How did LUCA make a living? Chemiosmosis in the origin of life. Bioessays **32**, 271–280 (2010). 20108228 10.1002/bies.200900131

[104] M. J. D. Russell, R. M. Hall, A. J. Sherringham, A hydrothermally precipitated catalytic iron sulphide membrane as a first step toward life. J. Mol. Evol. **39**, 231–243 (1994).

[105] D. S. K. Kelley , AT3-60 Shipboard party, an off-axis hydrothermal vent field near the Mid-Atlantic Ridge at 30 degrees N. Nature **412**, 145–149 (2001). 11449263 10.1038/35084000

[106] I. Halevy, A. Bachan, The geologic history of seawater pH. Science **355**, 1069–1071 (2017). 28280204 10.1126/science.aal4151

[107] G. MacLeod, C. McKeown, A. J. Hall, M. J. Russell, Hydrothermal and oceanic pH conditions of possible relevance to the origin of life. Orig. Life Evol. Biosphs. **24**, 19–41 (1994). 10.1007/BF01582037 11536657

[108] N. Lane, Proton gradients at the origin of life. Bioessays **39**, 1600217 (2017). 10.1002/bies.201600217 28503790

[109] N. Lane, W. F. Martin, The origin of membrane bioenergetics. Cell **151**, 1406–1416 (2012). 23260134 10.1016/j.cell.2012.11.050

[110] A. L. Ducluzeau , Was nitric oxide the first deep electron sink? Trends Biochem. Sci. **34**, 9–15 (2009). 19008107 10.1016/j.tibs.2008.10.005

[111] T. Namani, P. Walde, From decanoate micelles to decanoic acid/dodecylbenzenesulfonate vesicles. Langmuir **21**, 6210–6219 (2005). 15982022 10.1021/la047028z

[112] S. F. R. Jordan , Promotion of protocell self-assembly from mixed amphiphiles at the origin of life. Nat. Ecol. Evol. **3**, 1705–1714 (2019). 31686020 10.1038/s41559-019-1015-y

[113] Y. Koga, T. Kyuragi, M. Nishihara, N. Sone, Did archaeal and bacterial cells arise independently from noncellular precursors? A hypothesis stating that the advent of membrane phospholipid with enantiomeric glycerophosphate backbones caused the separation of the two lines of descent. J. Mol. Evol. **46**, 54–63 (1998). 9419225 10.1007/pl00006283

[114] A. Y. Mulkidjanian, K. S. Makarova, M. Y. Galperin, E. V. Koonin, Inventing the dynamo machine: The evolution of the F-type and V-type ATPases. Nat. Rev. Microbiol. **5**, 892–899 (2007). 17938630 10.1038/nrmicro1767

[115] J. Jablonska, D. S. Tawfik, The evolution of oxygen-utilizing enzymes suggests early biosphere oxygenation. Nat. Ecol. Evol. **5**, 442–448 (2021). 33633374 10.1038/s41559-020-01386-9

[116] F. Baymann , The redox protein construction kit: Pre-last universal common ancestor evolution of energy-conserving enzymes. Philos. Trans. R. Soc. Lond. B Biol. Sci. **358**, 267–274 (2003). 12594934 10.1098/rstb.2002.1184 PMC1693098

[117] Y. F. Ou , Expanding the phylogenetic distribution of cytochrome b-containing methanogenic archaea sheds light on the evolution of methanogenesis. ISME J. **16**, 2373–2387 (2022). 35810262 10.1038/s41396-022-01281-0 PMC9478090

[118] S. Basak, S. Nader, S. S. Mansy, Protometabolic reduction of NAD(+) with alpha-keto acids. JACS Au **1**, 371–374 (2021). 34467301 10.1021/jacsau.0c00124 PMC8395669

[119] D. P. Henriques Pereira , Role of geochemical protoenzymes (geozymes) in primordial metabolism: Specific abiotic hydride transfer by metals to the biological redox cofactor NAD(). FEBS J. **289**, 3148–3162 (2022). 34923745 10.1111/febs.16329 PMC9306933

[120] J. M. Weber , Iron-sulfur minerals drive NAD+ reduction under prebiotic Earth conditions. Astrobiology **22**, 25–34 (2022).34591607

[121] P. Dalai, N. Sahai, A model protometabolic pathway across protocell membranes assisted by photocatalytic minerals. J. Phys. Chem. B **124**, 1469–1477 (2020).

[122] C. G. Bonfio , Prebiotic iron–sulfur peptide catalysts generate a pH gradient across model membranes of late protocells. Nat. Catalysis **1**, 616–662 (2018).

[123] S. Dagar, S. Sarkar, S. Rajamani, Porphyrin in prebiotic catalysis: Ascertaining a route for the emergence of early Metalloporphyrins. Chembiochem **23**, e202200013 (2022). 35233914 10.1002/cbic.202200013

[124] M. Keller, E. Blochl, G. Wachtershauser, K. O. Stetter, Formation of amide bonds without a condensation agent and implications for origin of life. Nature **368**, 836–838 (1994). 8159243 10.1038/368836a0

[125] S. A. Sanden, R. Yi, M. Hara, S. E. McGlynn, Simultaneous synthesis of thioesters and iron-sulfur clusters in water: Two universal components of energy metabolism. Chem. Commun. (Camb). **56**, 11989–11992 (2020). 32896847 10.1039/d0cc04078a

[126] L. M. Barge , From chemical gardens to fuel cells: Generation of electrical potential and current across self-assembling iron mineral membranes. Angew. Chem. Int. Ed. Engl. **54**, 8184–8187 (2015). 25968422 10.1002/anie.201501663

[127] L. M. Barge , From chemical gardens to chemobrionics. Chem. Rev. **115**, 8652–8703 (2015). 26176351 10.1021/acs.chemrev.5b00014

[128] F. Glaab, J. Rieder, J. M. Garcia-Ruiz, W. Kunz, M. Kellermeier, Diffusion and precipitation processes in iron-based silica gardens. Phys. Chem. Chem. Phys. **18**, 24850–24858 (2016). 27397509 10.1039/c6cp02107g

[129] D. S. K. Kelley , A serpentinite-hosted ecosystem: The lost city hydrothermal field. Science **307**, 1428–1434 (2005). 15746419 10.1126/science.1102556

[130] Y. Li, N. Kitadai, R. Nakamura, Chemical diversity of metal sulfide minerals and its implications for the origin of life. Life (Basel) **8**, 46 (2018). 30308967 10.3390/life8040046 PMC6316247

[131] A. Y. Yamaguchi , Electrochemical CO2 reduction by Ni-containing iron sulfides: How is CO2 electrochemically reduced at bisulfide-bearing deep-sea hydrothermal precipitates? Electrochim. Acta **141**, 311–318 (2014).

[132] H. Baltscheffsky, "Energy conversion leading to the origin and early evolution of life: Did inorganic pyrophosphate precede adenosine triphosphate" in Origin and Evolution of Biological Energy Conversion, H. Baltscheffsky, Ed. (VCH Publishers, Cambridge, 1996), pp. 1–9.

[133] L. M. Barge , Pyrophosphate synthesis in iron mineral films and membranes simulating prebiotic submarine hydrothermal precipitate. Geochim. Cosmochim. Acta **128**, 1–12 (2014).

[134] I. I. M. de Zwart, S. J. Meade, A. J. Pratt, Biomimetic phosphoryl transfer catalysed by iron(II)-mineral precipitates. Geochim. Cosmochim. Acta **68**, 4093–4098 (2004).

[135] H.-J.B. Kim, S. A. Benner, Abiotic synthesis of nucleoside 5’-triphosphates with nickel borate and cyclic trimetaphosphate (CTMP). Astrobiology **21**, 298–306 (2021). 33533695 10.1089/ast.2020.2264

[136] G. Arrhenius, B. Sales, S. Mojzsis, T. Lee, Entropy and charge in molecular evolution–the case of phosphate. J. Theor. Biol. **187**, 503–522 (1997). 9299295 10.1006/jtbi.1996.0385

[137] Q. Wang, L. M. Barge, O. Steinbock, Microfluidic production of pyrophosphate catalyzed by mineral membranes with steep pH gradients. Chemistry **25**, 4732–4739 (2019). 30725519 10.1002/chem.201805950

[138] M. J. Russell, Green rust: The simple organizing ‘seed’ of all life? Life (Basel) **8**, 35 (2018). 30150570 10.3390/life8030035 PMC6161180

[139] M. J. Russell, W. Nitschke, E. Branscomb, The inevitable journey to being. Philos. Trans. R. Soc. Lond. B Biol. Sci. **368**, 20120254 (2013). 23754808 10.1098/rstb.2012.0254 PMC3685457

[140] E. J. R. Milner-White, M. J. Russell, Predicting peptide and protein conformations in early evolution. Biol. Direct **3**, 3 (2008). 18226248 10.1186/1745-6150-3-3 PMC2241844

[141] E. J. Milner-White, M. J. Russell, Polyphosphate-peptide synergy and the organic takeover at the emergence of life. J. Cosmol. **10**, 3217–3229 (2010).

[142] W. R. Nitschke, M. J. Russell, Hydrothermal focusing of chemical and chemiosmotic energy, supported by delivery of catalytic Fe, Ni, Mo/W Co, S and Se, forced life to emerge. J. Mol. Evol. **69**, 481–496 (2009). 19911220 10.1007/s00239-009-9289-3

[143] A. Lazcano, S. L. Miller, On the origin of metabolic pathways. J. Mol. Evol. **49**, 424–431 (1999). 10486000

[144] W. Nitschke , Aqueous electrochemistry: The toolbox for life’s emergence from redox disequilibria. Electrochem. Sci. Adv. **3**, e2100192 (2023).

[145] B. Schoepp-Cothenet , On the universal core of bioenergetics. Biochim. Biophys. Acta **1827**, 79–93 (2013). 22982447 10.1016/j.bbabio.2012.09.005

[146] S. G. Henao , Planetary minerals catalyze conversion of a polycyclic aromatic hydrocarbon to a prebiotic quinone: Implications for origins of life. Astrobiology **22**, 197–209 (2022). 35100015 10.1089/ast.2021.0024

