# Supplementary material for: Electron transport chains as a window into the earliest stages of evolution
Source: Proc Natl Acad Sci U S A. 2023 Aug 14;120(34):e2210924120. doi: 10.1073/pnas.2210924120 (PMC10451490; doi:10.1073/pnas.2210924120)
Supplement: Supplementary file 1 — Appendix 01 (PDF) [file pnas.2210924120.sapp.pdf]

The table below contains data used to determine the number of minimal LUCA proteome study predictions for each ETC component depicted in Figure 2 of the manuscript (Goldman *et al*., “Electron Transport Chains as a Window into the Earliest Stages of Evolution”). The columns are as follows:

1: The eggNOG cluster associated with an ETC Protein
2: The ETC protein or protein complex
3: The particular ETC that it is associated with
4: The number of LUCApedia database predictions associated with the eggNOG cluster (see Crapitto et al, 2022)
5-12: The individual LUCA proteome and whether or not they predict the eggNOG cluster to have LUCA ancestry

For each protein or protein complex, the subunit with the largest number of eggNOG predictions is used to determine the number of LUCA proteome predictions depicted in Figure 2 of the manuscript.

Proteins or subunits with no LUCApedia predictions are not shown below.

eggNOG cluster	Protein	System	Total	Delaye	Harris	Mirkin	Ranea	Srinivasan	Wang	Weiss	Yang
**COG0055**	ATP synthase	ATP synthase	3	+	-	+	+	-	-	-	-
**COG0056**	ATP synthase	ATP synthase	5	+	-	+	+	-	+	-	+
**COG0224**	ATP synthase	ATP synthase	3	+	-	+	+	-	-	-	-
**COG0355**	ATP synthase	ATP synthase	4	+	-	+	+	-	+	-	-
**COG0356**	ATP synthase	ATP synthase	2	-	-	+	-	-	+	-	-
**COG0636**	ATP synthase	ATP synthase	3	-	+	+	-	-	+	-	-
**COG0712**	ATP synthase	ATP synthase	2	+	-	+	-	-	-	-	-
**COG0843**	Cytochrome C oxidase	Oxidative Respiration	2	-	-	-	-	-	+	+	-
**COG1622**	Cytochrome C oxidase	Oxidative Respiration	1	-	-	-	-	-	+	-	-
**COG1845**	Cytochrome C oxidase	Oxidative Respiration	1	-	-	-	-	-	-	+	-
**COG2010**	Cytochrome C oxidase	Oxidative Respiration	1	-	-	-	-	-	+	-	-

**COG3278**	Cytochrome C oxidase	Oxidative Respiration	1	-	-	-	-	-	-	+	-
**COG0723**	Cytochrome BC1 Complex	Oxidative Respiration	3	-	-	+	-	-	+	+	-
**COG1290**	Cytochrome BC1 Complex	Oxidative Respiration	2	-	-	+	-	-	+	-	-
**COG2010**	Cytochrome BC1 Complex	Oxidative Respiration	1	-	-	-	-	-	+	-	-
**COG2857**	Cytochrome BC1 Complex	Oxidative Respiration	2	+	-	-	-	-	+	-	-
**COG3245**	Cytochrome C	Oxidative Respiration	1	-	-	-	-	-	+	-	-
**COG0377**	NADH dehydrogenase complex	Oxidative Respiration	1	-	-	+	-	-	-	-	-
**COG0446**	NADH dehydrogenase complex	Oxidative Respiration	5	+	-	+	-	+	+	-	+
**COG0649**	NADH dehydrogenase complex	Oxidative Respiration	1	-	-	+	-	-	-	-	-
**COG0655**	NADH dehydrogenase complex	Oxidative Respiration	1	-	-	-	-	-	+	-	-
**COG0778**	NADH dehydrogenase complex	Oxidative Respiration	4	+	-	+	-	-	+	+	-
**COG0838**	NADH dehydrogenase complex	Oxidative Respiration	1	+	-	-	-	-	-	-	-
**COG0852**	NADH dehydrogenase complex	Oxidative Respiration	1	-	-	+	-	-	-	-	-
**COG1009**	NADH dehydrogenase complex	Oxidative Respiration	1	-	-	-	-	-	-	+	-
**COG1034**	NADH dehydrogenase complex	Oxidative Respiration	2	+	-	+	-	-	-	-	-

**COG1143**	NADH dehydrogenase complex	Oxidative Respiration	2	-	-	+	-	-	-	+	-
**COG1249**	NADH dehydrogenase complex	Oxidative Respiration	6	+	-	+	+	+	+	-	+
**COG1252**	NADH dehydrogenase complex	Oxidative Respiration	1	+	-	-	-	-	-	-	-
**COG1894**	NADH dehydrogenase complex	Oxidative Respiration	1	+	-	-	-	-	-	-	-
**COG1979**	NADH dehydrogenase complex	Oxidative Respiration	1	-	-	-	-	-	+	-	-
**COG3634**	NADH dehydrogenase complex	Oxidative Respiration	1	+	-	-	-	-	-	-	-
**COG0479**	succinate dehydrogenase complex	Oxidative Respiration	3	+	-	+	-	+	-	-	-
**COG1053**	succinate dehydrogenase complex	Oxidative Respiration	4	-	-	+	-	+	+	-	+
**COG2009**	succinate dehydrogenase complex	Oxidative Respiration	2	-	-	+	-	+	-	-	-
**COG2142**	succinate dehydrogenase complex	Oxidative Respiration	1	-	-	-	-	+	-	-	-
**COG3029**	succinate dehydrogenase complex	Oxidative Respiration	1	-	-	-	-	+	-	-	-
**COG0723**	CytochromeB6F Complex	Oxygenic Photosynthesis	3	-	-	+	-	-	+	+	-
**COG0739**	CytochromeB6F Complex	Oxygenic Photosynthesis	3	+	-	-	-	+	+	-	-
**COG1290**	CytochromeB6F Complex	Oxygenic Photosynthesis	2	-	-	+	-	-	+	-	-

**COG0369**	FNR	Oxygenic Photosynthesis	3	+	-	-	-	+	+	-	-
**COG0492**	FNR	Oxygenic Photosynthesis	6	+	+	+	-	-	+	+	+
**COG0237**	Photosystem1	Oxygenic Photosynthesis	4	-	-	+	-	+	+	-	+
**COG0448**	Photosystem1	Oxygenic Photosynthesis	2	+	-	-	-	-	+	-	-
**COG0457**	Photosystem1	Oxygenic Photosynthesis	3	+	-	+	-	+	-	-	-
**COG1143**	Photosystem1	Oxygenic Photosynthesis	2	-	-	+	-	-	-	+	-
**COG2885**	Photosystem1	Oxygenic Photosynthesis	2	-	-	-	-	+	+	-	-
**COG1555**	Photosystem2	Oxygenic Photosynthesis	2	+	-	-	-	-	+	-	-
**COG0852**	MBH	Methanogenic ETC *Methano-microbiales*	1	-	-	+	-	-	-	-	-
**COG1143**	MBH	Methanogenic ETC *Methano-microbiales*	2	-	-	+	-	-	-	+	-
**COG1863**	MBH	Methanogenic ETC *Methano-microbiales*	1	-	-	-	-	+	-	-	-
**COG3260**	MBH	Methanogenic ETC *Methano-microbiales*	1	+	-	-	-	-	-	-	-
**arCOG01548/ COG0852**	FPO	Methanogenic ETC *M. acetivorans*	1	+	-	-	-	-	-	-	-
**COG1245**	Rnf complex	Acetogenesis *A. woodii*	1	+	-	-	-	-	-	-	-
**COG2878**	Rnf complex	Acetogenesis *A. woodii*	1	+	-	-	-	-	-	-	-
**COG4658**	Rnf complex	Acetogenesis *A. woodii*	1	-	-	+	-	-	-	-	-
**COG4660**	Rnf complex	Acetogenesis *A. woodii*	2	-	+	+	-	-	-	-	-

<u>References:</u>

Crapitto, A.J., Campbell, A., Harris, A.J., Goldman, A.D. (2022) A consensus view of the proteome of the last universal common ancestor. Ecol Evol. 12(6), e8930.

Delaye, L., Becerra, A., & Lazcano, A. (2005). The last common ancestor: What's in a name? Origins of Life and Evolution of Biospheres, 35, 537–554.

Harris, J. K., Kelley, S. T., Spiegelman, G. B., & Pace, N. R. (2003). The genetic core of the universal ancestor. Genome Research, 13, 407.

Mirkin, B. G., Fenner, T. I., Galperin, M. Y., & Koonin, E. V. (2003). Algorithms for computing parsimonious evolutionary scenarios for genome evolution, the last universal common ancestor and dominance of horizontal gene transfer in the evolution of prokaryotes. BMC Evolutionary Biology, 3, 2.

Ranea, J. A., Sillero, A., Thornton, J. M., & Orengo, C. A. (2006). Protein superfamily evolution and the last universal common ancestor (LUCA). Journal of Molecular Evolution, 63, 513–525.

Srinvasan, V., & Morowitz, H. J. (2009). The canonical network of autotrophic intermediary metabolism: minimal metabolome of a reductive chemoautotroph. Biological Bulletin, 216, 126–130.

Wang, M., Yafremava, L. S., Caetano-Anollés, D., Mittenthal, J. E., & Caetano-Anollés, G. (2007). Reductive evolution of architectural repertoires in proteomes and the birth of the tripartite world. Genome Research, 17, 1572–1585.

Weiss, M. C., Sousa, F. L., Mrnjavac, N., Neukirchen, S., Roettger, M., Nelson-Sathi, S., & Martin, W. F. (2016). The physiology and habitat of the last universal common ancestor. Nature Microbiology, 1, 16116.

Yang, S., Doolittle, R. F., & Bourne, P. E. (2005). Phylogeny determined by protein domain content. Proceedings of the National Academy of Sciences, 102(2), 373–378.
